# A Versatile‐Designable Framework for Active and Programmable Shape‐Morphing Soft Matter Systems: From Inverse Design to Closed‐Loop Control

**DOI:** 10.1002/advs.76241

**Published:** 2026-06-22

**Authors:** Kai Liu, Peiling Xie, Ruitong Song, Banghan Liu, Rui Guo, Jiu‐an Lv

**Affiliations:** ^1^ School of Materials Science and Engineering Zhejiang University Hangzhou China; ^2^ Key Laboratory of 3D Micro/Nano Fabrication and Characterization of Zhejiang Province School of Engineering Westlake University Hangzhou Zhejiang Province China; ^3^ Institute of Advanced Technology Westlake Institute for Advanced Study Hangzhou Zhejiang Province China

**Keywords:** closed‐loop control, deep learning, inverse design, liquid crystal elastomers, programmable morphing, soft matter

## Abstract

Developing soft matter systems with programmability, multifunctional integration, and environmental adaptability represents a critical challenge in soft robotics and smart materials. Herein, we propose a versatile framework for constructing active and programmable shape‐morphing soft matter systems based on addressable actuation and strain‐constraint mechanisms. Using liquid crystal elastomers (LCEs) and conductive constraint strips serving simultaneously as geometric constraints and localized Joule heaters, this framework circumvents complex microstructural manipulation, enabling addressable electrothermal actuation and deterministic 2D‐to‐3D morphological transformation. Combined with the established analytical model, we propose an inverse design strategy capable of reconstructing complex target surfaces featuring spatially non‐uniform curvatures. To demonstrate its integration capability, we incorporate shape memory polymers (SMPs) and crack‐based sensors via a thermally decoupled design to construct a proprioceptive lockable soft robotic system (PLSRS), exhibiting zero‐energy shape retention and real‐time proprioception. Finally, we validate this system by deploying the PLSRS in a flapping‐wing robot, where a 1D convolutional neural network (1D‐CNN) optimized via the grey wolf optimizer (GWO) deciphers aeroelastic signals to estimate wind speed and trigger autonomous adaptive wing regulation. This work successfully fuses physical intelligence with computational intelligence, providing a versatile platform for next‐generation adaptive soft robots.

## Graphical abstract

A versatile framework integrates addressable electrothermal actuation and strain‐constraint mechanisms to construct programmable shape‐morphing soft matter systems. By combining an analytical inverse design strategy for high‐fidelity 3D surface reconstruction with deep learning‐based closed‐loop control, this approach enables zero‐energy shape locking, real‐time proprioception, and autonomous environmental adaptation in soft robots.

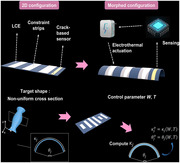

## 1 Introduction

Inspired by the ability of biological organisms to adapt to complex environments through morphological transformation—for instance, pine cones opening their scales in response to humidity changes [1]—developing intelligent systems capable of autonomous deformation has become a core pursuit in the fields of soft robotics and smart materials [2]. Although passive mechanical structures (e.g., pneumatic networks [3, 4, 5], origami/kirigami [6, 7, 8], mechanically guided 3D assembly [9, 10, 11], and peeling‐induced shape morphing [12]) offer extensive geometric design freedom, they are often limited by bulky tethering systems and restricted mechanical robustness. Consequently, stimuli‐responsive smart materials—including polymer gels [13, 14, 15, 16], shape memory polymers (SMPs) [17, 18, 19], and liquid crystal elastomers (LCEs) [20, 21, 22, 23, 24, 25, 26, 27, 28]—have emerged as premier candidates for high‐performance soft robots due to their intrinsic compliance, large deformation capabilities, or high energy densities [29], and have been widely utilized to develop soft actuators capable of complex, reversible shape transformations and programmable behaviors [30, 31, 32]. However, transforming these materials from basic deformation units into active and programmable shape‐morphing soft matter systems faces a critical challenge of the complexity of deformation programming. Existing strategies typically rely on precise anisotropic manipulation of the material's microstructure, such as gradient crosslinking in polymer gels [33, 34] or complex mesogen alignment in LCEs [35, 36, 37]. While effective, these programming methods are often limited by cumbersome fabrication processes, high costs, and significant barriers to large‐scale system integration.

To circumvent these limitations, a macrostructural programming approach based on external structural heterogeneity has been leveraged. This approach takes advantage of macroscopic geometric constraints or stiffness gradients to guide material deformation, effectively circumventing the complexity of microscopic processing [38, 39, 40]. In this approach, LCEs, with their significant anisotropic thermal contraction, serve as an ideal model material to validate the macrostructural programming strategy. In particular, the strain‐constraint strategy, which induces deformation via strain mismatch, has demonstrated great potential [41, 42, 43]. Despite simplifying fabrication, current implementations remain limited by actuation mechanisms (often relying on global heating or external light sources [41, 42, 43, 44]) and functional singularity (lacking sensing and closed‐loop control [45, 46]), hindering those systems to meet the demands in addressable control and environmental adaptability for intelligent soft robotics.

Herein, we propose a versatile framework for constructing active and programmable shape‐morphing soft matter systems based on addressable actuation and strain‐constraint mechanisms. Utilizing conductive strips as dual‐functional elements (serving as geometric constraints and localized Joule heaters) and using LCEs as a model material, we achieve deterministic 2D‐to‐3D morphological transformation and addressable actuation without the need for complex microstructural manipulation. This framework not only achieves high‐fidelity reconstruction of complex surfaces via an inverse design strategy but, more importantly, offers an open architecture that seamlessly integrates SMPs and crack‐based sensors to construct a proprioceptive lockable soft robotic system (PLSRS) equipped with proprioception and zero‐energy locking capabilities. This system features thermally decoupled actuation, zero‐energy shape retention, and real‐time kinesthetic feedback. Finally, as a demonstration of the fusion of physical intelligence (structural morphing, locking, and proprioception) and computational intelligence (intelligent perception and optimization), we deploy the PLSRS in a flapping‐wing robot. Assisted by a 1D convolutional neural network (1D‐CNN) optimized via the grey wolf optimizer (GWO), the system deciphers aeroelastic signals to estimate wind speed and autonomously regulates wing morphology, establishing a versatile platform for next‐generation intelligent soft robots.

## 2 Results and Discussion

### 2.1 Active and Programmable Shape‐Morphing Soft Matter System With Integrated Sensing and Closed‐Loop Control

**FIGURE 1:**
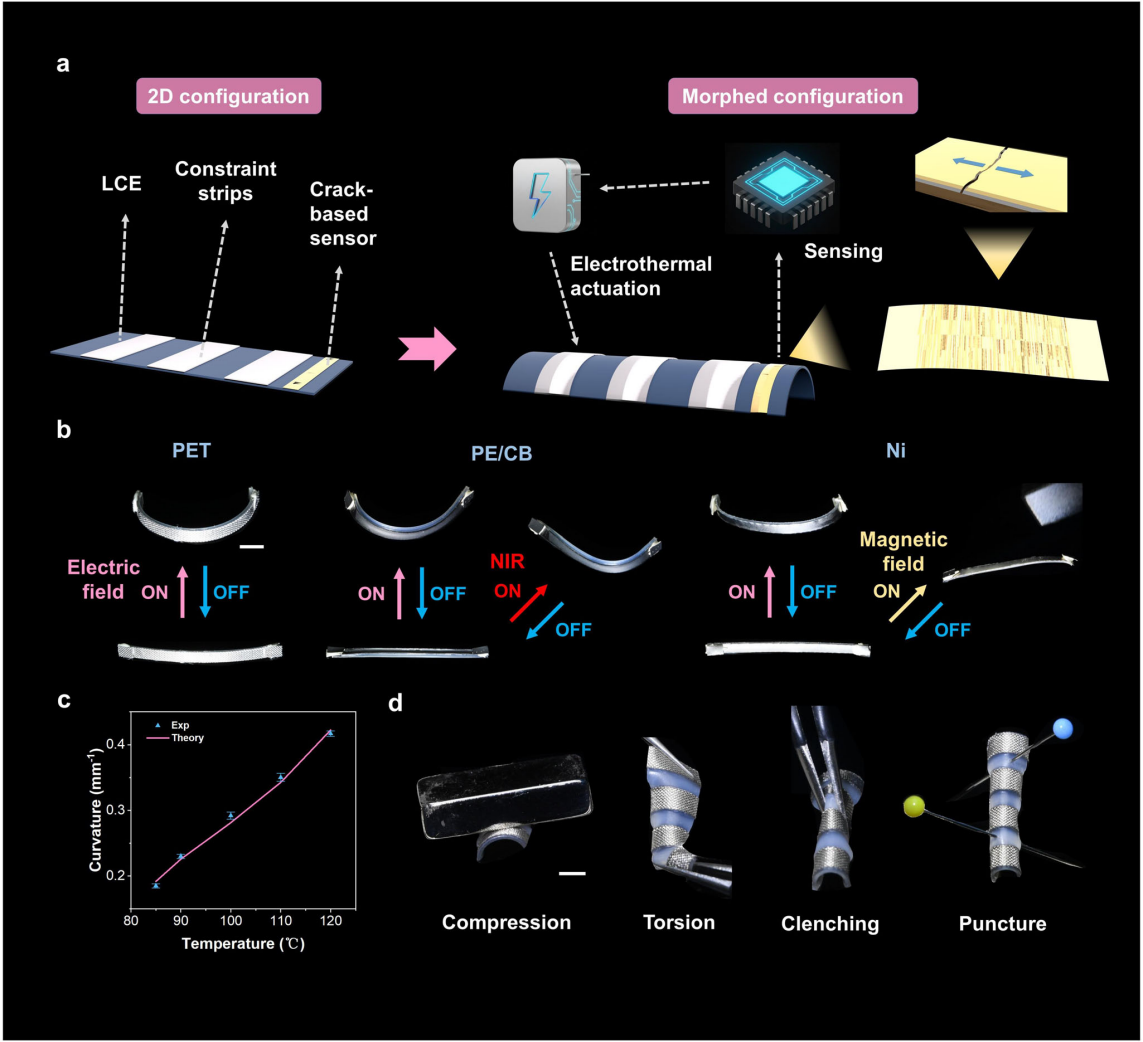
3D morphing system based on strain‐constraint mechanism. (a) Schematic illustration of the integrated framework and the morphing mechanism driven by strain constraint. (b) Material universality demonstrated by various functional constraint strips. Scale bars: 4 mm. (c) Experimental verification of the analytical model regarding the relationship between curvature and temperature. Data are presented as mean ± SD (*N* = 3). (d) Mechanical robustness of the morphing structure under compression, torsion, clenching, and puncture. These demonstrations were consistently verified across *N* = 3 independent samples. Scale bars: 4 mm.

As shown in Figure 1a, we propose an active and programmable shape‐morphing soft matter system that integrates actuation, programming, and sensing into a single unit. This system seamlessly integrates an anisotropic LCE matrix, conductive strips serving as both constraints and addressable local heaters, and crack‐based sensors within a monolithic soft structure. This integrated architecture not only triggers programmable 2D‐to‐3D morphological transformation via the electrothermal actuation of the conductive strips but also utilizes the crack‐based sensor to monitor morphing shapes in real‐time, establishing an actuation‐sensing‐feedback closed‐loop control system.

The core shape‐morphing mechanism of the system is based on strain‐constraint principle: when voltage is applied, the Joule heat generated by constraint strips induces programmable contraction of the LCE, while the strips themselves remain geometrically stable. This interlayer strain mismatch induced by the temperature field, coupled with the significant elastic modulus contrast, drives the planar structure to morph, thereby achieving directional shape transformation (Movie S1). As shown in Figure 1a, when all constraint strips are aligned parallel, the deformed structure presents a cylindrical shape defined by principal curvature *κ*. To quantitatively elucidate this mechanical mechanism, we established an analytical model (Text S1, Supporting Information). The principal curvature *κ* of the cylindrical surface can be expressed as:

$$\kappa =\frac{6EWl}{h_{s}}\frac{h\left(1+h\right)\left(T-T_{0}\right)\left(\alpha_{r}-\alpha_{s}\right)}{1+2EWlh\left(2+3h+2h^{2}\right)+E^{2}W^{2}l^{2}h^{4}}$$

where $W=\frac{W_{r}}{W_{s}}$, $l=\frac{l_{r}}{l_{s}}$, $h=\frac{h_{r}}{h_{s}}$, and $E=\frac{E_{r}\;\left(1-\upsilon_{s}^{2}\right)}{E_{s}\;\left(1-\upsilon_{r}^{2}\right)}$. *W*, *l*, *h*, *E*, *T*, *T_0_*, and *α* denote width, length, thickness, Young's modulus, actuation temperature, reference room temperature (25°C), and coefficient of thermal expansion, respectively (Figure S1, Supporting Information). Subscripts *s* and *r* correspond to the substrate and constraint strips, respectively.

Figure 1c illustrates the relationship between the principal curvature *κ* of the 3D structure and the actuation temperature of the LCE. The low modulus (∼17 MPa) of the LCE and the high modulus of the constraint strips (∼1.5 GPa) form a significant stiffness gap (Figure S2), which serves as the physical basis for effective strain constraint. The experimental sample dimensions were designed as follows: LCE substrate 24 × 8 mm, constraint strips 8 × 3 mm, with a spacing of 3 mm. The experimental results agree well with theoretical predictions, validating the accuracy of the analytical model. Furthermore, Figure S3 shows how the relative thickness modulates the deformation curvature.

Notably, this strain‐constraint strategy exhibits broad material universality. Beyond the conductive polyester (PET) fiber woven fabrics used in the initial system, we confirmed that various functional conductive materials can effectively induce similar morphing behaviors and endow the system with additional multimodal response capabilities (Figure 1b). For instance, polyethylene (PE)/carbon black (CB) films as constraint strips not only support Joule heating actuation but also introduce near‐infrared light response characteristics (Movie S2); meanwhile, nickel (Ni) film constraint strips are compatible with magnetic response actuation modes (Movie S3). This flexibility in material selection significantly broadens the application scenarios of this programmable morphing framework. Fundamentally, as established in our theoretical model, the morphing curvature is strictly governed by the effective modulus ratio (*E*) and the difference in coefficients of thermal expansion (*α_r_* − *α_s_*). This physical mechanism theoretically allows the framework to adapt to diverse material combinations exhibiting distinct mechanical contrast and strain mismatch, thereby inherently endowing the strategy with broad material universality. Furthermore, enabled by an all‐soft structural design, our system overcomes the limitations associated with conventional rigid or brittle architectures and exhibits high mechanical robustness (Figure 1d). The 3D structure maintains excellent resilience under diverse mechanical deformations, including compression, torsion, clenching, and even puncture by sharp objects. Peel strength tests confirmed the formation of a robust interfacial bond between the LCE and the constraint strips (Figure S4). Furthermore, a 200 000‐s cyclic actuation test showed a vertical displacement degradation of less than 3%, indicating outstanding fatigue resistance (Figure S5; see Figure S6 for basic LCE actuation performance).

### 2.2 Geometric Programming and Multimodal Reconfiguration of Complex 3D Surfaces

**FIGURE 2:**
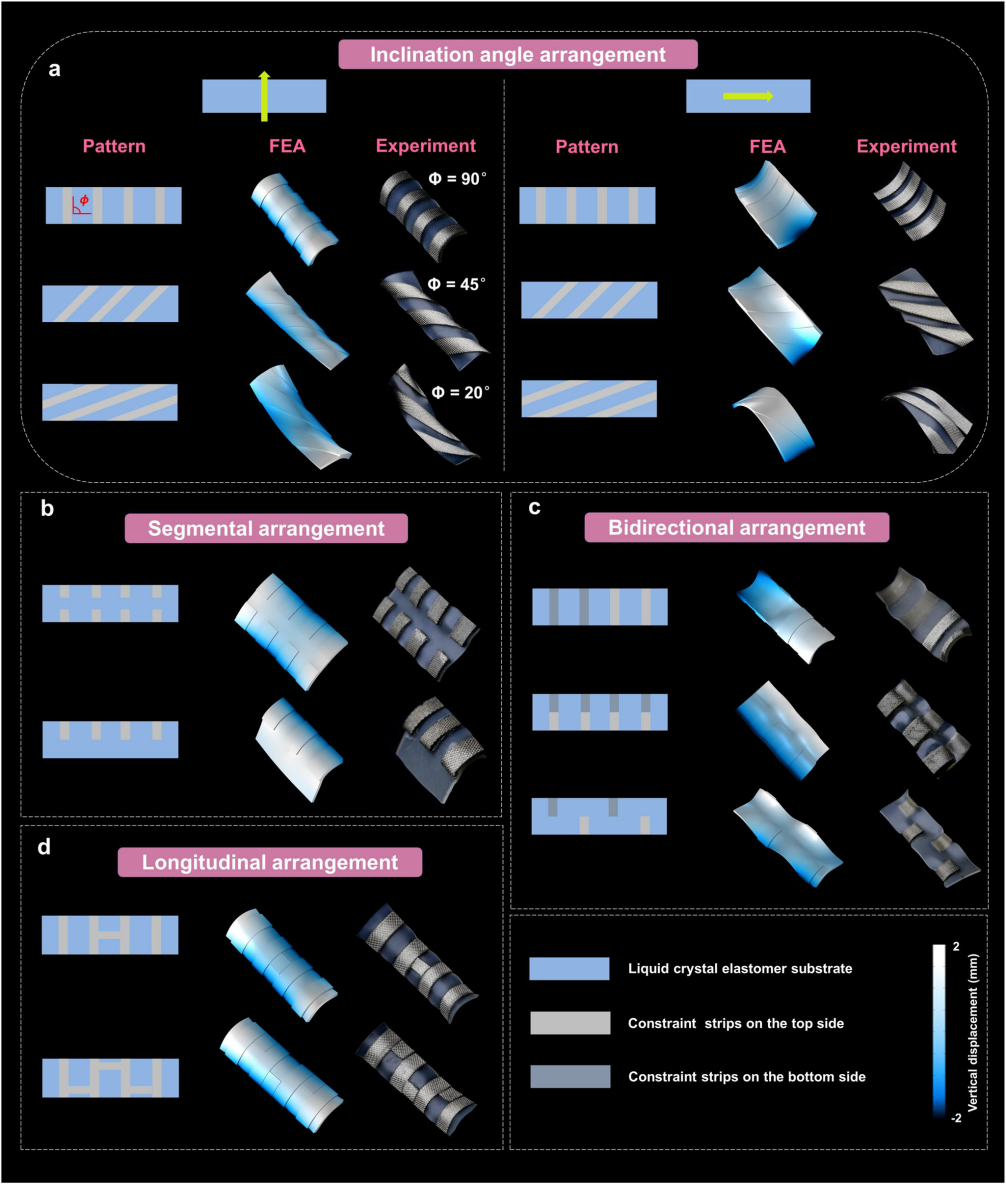
Geometric programming of complex 3D surfaces. (a) Shape variations regulated by the inclination angle of constraint strips and the orientation of the LCE. The green arrows denote the orientation of the liquid crystal mesogens within the LCE substrate. (b) Local curvature modulation based on spatially non‐uniform constraints. (c) Complex surfaces enabled by double‐sided constraints. (d) Construction of non‐zero Gaussian curvature surfaces via biaxial strain mismatch.

Based on the aforementioned strain‐constraint mechanism, we achieved the deterministic generation of complex 3D shapes through the discretized design of the conductive constraint strip layout. Figure 2 displays various representative constraint strip layouts and their corresponding 3D morphologies. The experimental results demonstrate a high degree of agreement with Finite Element Analysis (FEA) simulations, validating the precision of this design strategy. Figure 2a indicates that by adjusting the inclination angle *ϕ* of the constraint strips relative to the LCE long axis and the orientation of the LCE, different 3D shape variations can be achieved. Variations in the LCE orientation direction led to curvature reversal, while a decrease in *ϕ* results in the emergence of non‐cylindrical 3D shapes.

Furthermore, we introduced spatially non‐uniform constraint as a key geometric design parameter. As shown in Figure 2b, when constraint strips are designed to locally cover only a portion of the LCE width, a significant strain gradient forms between the constrained and free regions. This difference in strain field distribution, induced by the spatial truncation of the constraint layout, directly leads to the spatial modulation of local curvature. This strategy breaks the deformation uniformity along the width direction, providing a flexible means of regulation for designing complex surfaces with non‐uniform curvature distributions. On this basis, leveraging the double‐sided processing capability of LCEs allows for further construction of complex 3D shapes. As shown in Figure 2c, by alternately arranging constraint strips on both sides of the substrate, we not only achieved a smooth spatial transition from negative to positive curvature and sinusoidal wave‐like surfaces but also constructed unit tessellation structures featuring alternating positive and negative principal curvatures. Most importantly, to break the geometric limitations dominated by uniaxial constraints and actively construct doubly curved surfaces with non‐zero Gaussian curvature, we introduced a biaxial orthogonal constraint strategy. As shown in Figure 2d, by simultaneously applying constraints along both the long and short axes of the LCE, forcing the material to undergo biaxial strain mismatch, we successfully constructed saddle‐shaped structures with negative Gaussian curvature as well as tessellated surfaces integrating mixed curvature units. Demonstrating the versatility of this approach, the system can construct not only periodic structures but also extend to more complex surfaces with spatially varying curvature characteristics, such as a human face profile (Figure S7a).

Beyond static geometric design, the system's unique electrothermal actuation mechanism endows it with one‐to‐many reconfigurability. Benefiting from the independent addressing capability of the constraint strips, we can trigger various target shapes (such as diverse vase profiles, see Figure S7b) on the same planar structure by establishing a non‐uniform temperature field. Furthermore, the geometric dimensions (e.g., width) of the constraint strips introduce a coupling mechanism between mechanical constraint and electrothermal effects. As shown in Figure S8a and Movie S4, narrower strips exhibit larger bending curvature under the same current. This stems from the synergistic effect of their weaker mechanical constraint and higher Joule heating power density. Based on this principle, combined with the design of the LCE orientation direction, we realized complex torsional actuation (Figure S8b and Movie S5) and the morphing of bio‐inspired flowers (Figure S8c).

### 2.3 Inverse Design Strategy

**FIGURE 3:**
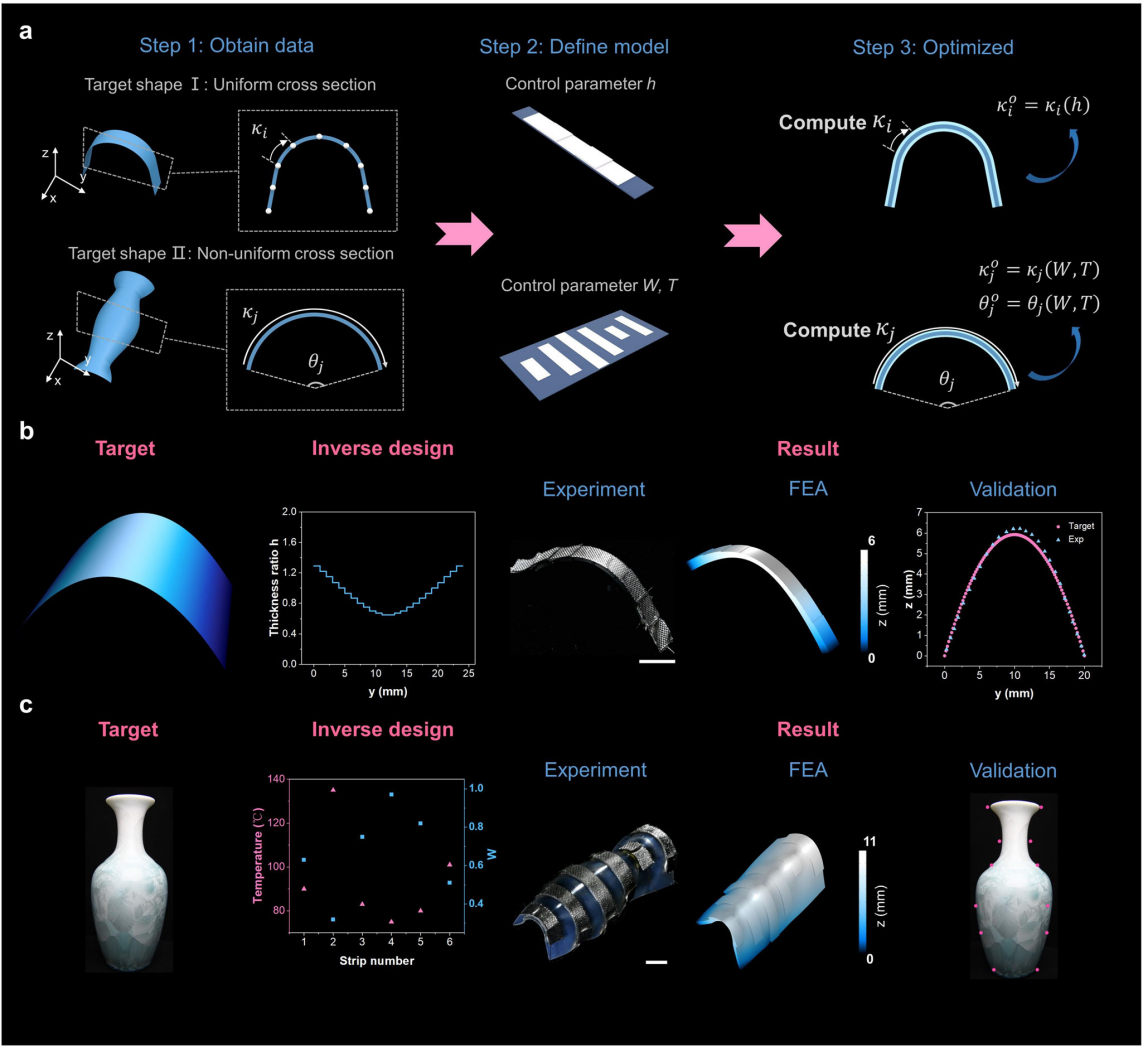
Inverse design of 3D surfaces. (a) Flowchart of the inverse design strategy converting target shapes into fabrication parameters. The optimization processes for different target shapes are conducted independently. The subscripts *i* and *j* refer to the *i*‐th and *j*‐th discrete segments in space. (b) Reconstruction of a surface with a spatially non‐uniform curvature distribution along the y‐axis. Scale bars: 6 mm. (c) Reconstruction of a vase profile via joint optimization of constraint strip width and temperature. The strip numbers 1 to 6 denote the constraint segments arranged from top to bottom along the LCE film. Scale bars: 4 mm.

Based on the quantitative mapping relationship established by the aforementioned analytical model between geometric parameters and deformation metrics, we propose an intuitive inverse design strategy (Figure 3a). This strategy aims to solve the inverse problem of deriving the 2D planar precursor geometric parameters and actuation conditions (temperature *T*) from a target 3D surface. We transform this process into a constrained non‐linear least‐squares optimization problem. A spatial discretization strategy is adopted to divide the target surface along the axis into several equidistant discrete segments, extracting target curvature $\kappa_{i,j}^{o}$ and central angle $\theta_{i,j}^{o}$ for each segment, where the subscripts *i* and *j* denote the spatial indices of these discrete segments across the 2D domain of a single target surface. To eliminate the potential error bias caused by the magnitude mismatch between these two target variables, the optimization model is formulated to minimize the sum of squared relative residuals between the predicted and target values:

$$\begin{array}{ccc} minf\left(W,h,T\right) & = & \sum_{i}\sum_{j}{\left[\frac{\kappa \left(W,h,T\right)-\kappa_{i,j}^{o}}{\kappa_{i,j}^{o}}\right]}^{2}\\ & & +{\left[\frac{\theta \left(W,h,T\right)-\theta_{i,j}^{o}}{\theta_{i,j}^{o}}\right]}^{2} \end{array}$$

subject to the physical and geometric constraints: 0 < *W* ≤ 1, 0 < *h*, and *T_min_* ≤ *T* ≤ *T_max_*. Here, *T_min_ and T_max_* represent the onset and end temperature of the LCE's actuation, respectively. The variables are selected from the available design parameters (i.e., width ratio *W*, thickness ratio *h*, and actuation temperature *T*) depending on the specific target. This non‐linear optimization is numerically solved using the FindMinimum function of Wolfram Mathematica. Depending on the geometric complexity of target surface, this framework supports either a simple decoupled optimization (tuning a single variable while fixing others) or a joint optimization (simultaneously tuning multiple coupled variables).

For 3D surfaces with a spatially non‐uniform curvature distribution along the *y*‐axis, we utilized a simple decoupled optimization, selecting the thickness ratio *h* as the sole design variable to tune the local bending stiffness and strain constraint efficacy. By calculating the average target curvature of each discrete segment along the *y*‐axis and solving the aforementioned optimization model, we obtained the spatial distribution function of the thickness ratio (Figure 3b). To evaluate the repeatability of the experimental validation, we fabricated four independent samples (*N* = 4) using the optimized design parameters. As shown in Figure S9, the morphing profiles of all samples exhibit high consistency and highly align with the target profile, yielding an average mean absolute error (MAE) of 199 µm. This result confirms the capability and good repeatability of this strategy to achieve high‐fidelity shape morphing through the fine‐tuning of thickness parameters.

For complex 3D shapes with uniform curvature along the *y*‐axis but non‐uniform cross‐sectional (along the *x*‐axis) profiles (such as vase structures), we adopted a joint optimization strategy of width ratio *W* and actuation temperature *T*. As shown in Figure 3c, we first extracted the geometric features of the target profile, converting the arc length *s_j_* and chord length *d_j_* of the *j*‐th cross‐section into the target curvature $\kappa_{j}^{o}$ and central angle $\theta_{j}^{o}$. Subsequently, the optimization model was solved to obtain the optimal width ratio *W* and temperature *T* for each discrete segment. To further evaluate the robustness of the optimization, perturbation analysis was performed around the optimum. As shown in Figure S10, the contour plot of the objective function exhibits a smooth valley‐like topography, indicating local stability against minor manufacturing tolerances or temperature fluctuations. Moreover, the tilted elliptical contours mathematically visualize the coupling between *W* and *T*. To validate the shape morphing, we employed a 2D projection method. The spatial distance between the two projected endpoints of each bent segment (i.e., the chord length) is strictly determined by its local curvature and central angle. As shown in Figure 3c, the 6 constrained segments generate 12 projected endpoints. The alignment of these 12 projected endpoints with the boundary profile of the target vase demonstrates that the fabricated LCE structure achieves the target configuration at each specific position after actuation (Table S1), validating the effectiveness of this inverse design strategy in handling multivariable coupled design problems.

While the proposed inverse design strategy demonstrates high fidelity in reconstructing complex 3D surfaces, the selection of design variables and its application boundaries merit further discussion. First, regarding the selection of design variables, although the theoretical framework supports multivariable optimization, we intentionally limited the number of simultaneously optimized variables based on a comprehensive consideration of mathematical well‐posedness and manufacturing convenience. This dimensionality reduction prevents the underdetermined system from generating non‐unique solutions and avoids complex operations. Consequently, the current combination of variables strikes an optimal balance, achieving high‐fidelity complex surface reconstruction without compromising the simplicity and reliability of macroscopic manufacturing. Second, the application of the inverse design strategy is primarily tailored for deterministic morphological programming under load‐free conditions. For applications involving simple global bending (such as the shape‐morphing system in Section 2.4), direct forward control is more efficient. Furthermore, when deployed in dynamic environments with external non‐linear aerodynamic loads (such as the flapping‐wing robot in Section 2.5), the static model becomes insufficient, which naturally necessitates the transition to deep learning‐based dynamic environmental perception and adaptive control.

### 2.4 Proprioceptive and Lockable Soft Robotic System via Multi‐Material Integration

To endow active and programmable shape‐morphing soft matters with synergistic capabilities of sensing, actuation, and stiffness tuning similar to biological muscles, we propose a proprioceptive lockable soft robotic system (PLSRS) integrating proprioception and shape‐locking functions.

**FIGURE 4:**
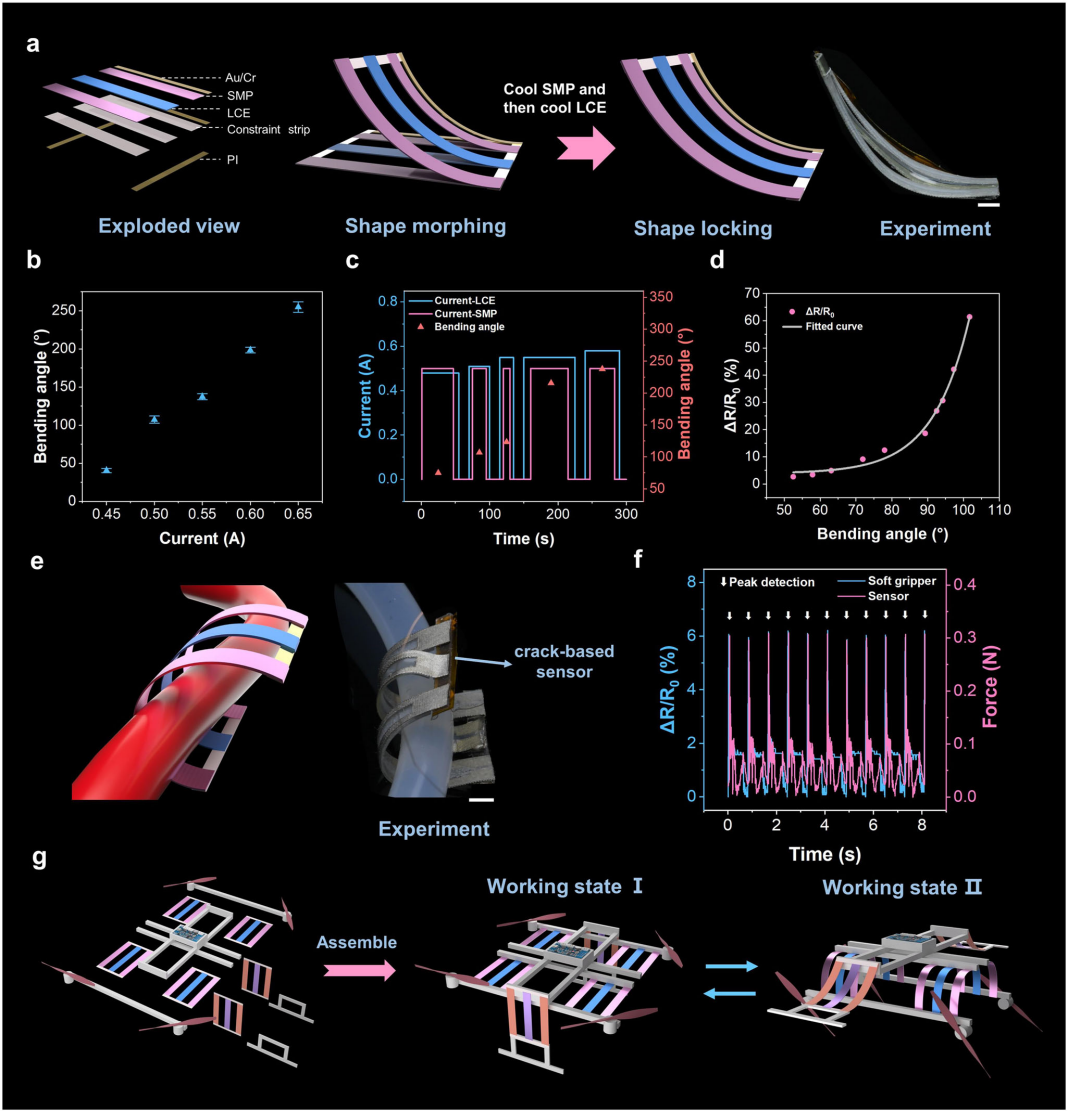
Design and application of the PLSRS. (a) Spatially decoupled multi‐material architecture integrating LCE, SMP, and crack‐based sensor. Scale bars: 7 mm. (b) Precise control of the PLSRS bending angle via current regulation. Data are presented as mean ± SD (*N* = 3). (c) Time‐variant response curves of the PLSRS during the multi‐stage locking process. (d) Electrical signal variations of the PLSRS during bending. e) Schematic illustration and photograph of the PLSRS used for bio‐monitoring. Scale bars: 7 mm. (f) Real‐time monitoring of pulse signals. (g) Schematic illustration of multimodal reconfiguration of a land‐air amphibious robot enabled by PLSRS.

This system adopts a spatially decoupled multi‐material integrated architecture, incorporating separated LCE actuation strips, SMP variable‐stiffness strips, and crack‐based sensors arranged side‐by‐side within a hollow isolation structure (Figure 4a). To ensure both internal functional independence and external application safety, we implemented a comprehensive 3D thermal decoupling strategy. Internally, to prevent thermal crosstalk, FEA confirms that the air gaps within this architecture act as effective thermal barriers between the functional layers (Figure S11). Externally, to prevent thermal transfer and potential thermal softening of the attached lightweight and soft structures, a dual‐protection mechanism was introduced. First, the conductive constraint strips were intentionally widened at both attachment ends to reduce local electrical resistance and Joule heating power density (Figure S12a). Second, thermal insulation layers (such as polyimide or foam tape) were introduced at the attachment interfaces, while an air gap was maintained between the middle actuating section and the attached structure. Infrared thermal imaging results (Figure S12b–d) demonstrate that even when the central actuating region of the PLSRS reaches 120°C, the surface temperature of the attached material remains at a safe 42°C, thereby ensuring its mechanical performance and structural stability (Figure S12e). Enabled by this systematic thermally decoupled design, this architecture achieves independent control of actuation and stiffness tuning while simultaneously endowing the system with real‐time proprioception capabilities. The core advantage of the PLSRS lies in its ability to achieve zero‐energy locking of deformed configurations via the phase transition of the SMP. Specifically, leveraging the significant modulus switching of the SMP around its glass transition temperature (*Tg*), we established the following control logic: First, the SMP layer is heated to *T_SMP_* > *Tg* to soften it (Figure S13a,b), releasing the mechanical constraint on the LCE; subsequently, the actuation strain is precisely regulated by independently controlling the Joule heating power of the LCE (Figure 4b and Figure S13d); once the target configuration is reached, the heating voltage across the SMP is removed, allowing it to naturally cool to room temperature (*T_R_* < *Tg*). At this stage, the SMP transitions back to the glassy state and stiffens substantially, utilizing its high stiffness to effectively overcome the elastic recoil stress of the LCE (Figure S13c), thereby locking the structure in the deformed state (Movie S6). To quantitatively evaluate this efficiency advantage, we compared the total energy consumption required to maintain a 72° bending angle for 1 h between the two modes. The conventional LCE required continuous heating to counteract elastic recoil, consuming as much as 2520 J; in contrast, the PLSRS consumed only 94.5 J during the actuation and locking phases, after which it maintained the shape in a power‐off state. This significant difference corresponds to an energy reduction of approximately 96.2%, resolving the high energy cost bottleneck of soft robotic systems in static holding tasks. The time‐variant response curves in Figure 4c further demonstrate that by synergistically controlling the thermal cycles of the LCE and SMP, the PLSRS can achieve continuous shape programming and multi‐stage locking.

The integrated crack‐based sensor (thickness ∼7.5 µm, mass ∼4.3 mg), composed of a metal layer deposited on a polyimide substrate [47], endows the PLSRS with mechanical perception capabilities similar to biological muscles while adding negligible stiffness burden to the structure. Based on the disconnection‐reconnection mechanism of metal nanocracks under tension/compression (Figure S14a), the sensor exhibits high electromechanical sensitivity. As shown in Figure 4d, variations in crack gaps with bending angles lead to reproducible and significant changes in resistance, enabling real‐time and precise feedback on the bending state of the PLSRS. Dynamic characterization reveals that the sensor exhibits a high sensitivity of 0.53% current change per degree and a low hysteresis of less than 1.16% (Figure S14b), ensuring high repeatability. Furthermore, the sensor demonstrates a rapid dynamic response with a rise time of only 57 ms (Figure S14c), sufficient to capture fast deformations. Collectively, these performance metrics provide reliable signal feedback for closed‐loop control.

Benefiting from the shape‐locking capability and the high sensitivity of the crack‐based sensor to minute vibrations, the PLSRS demonstrates unique advantages in biological physiological signal monitoring. We simulated a human arterial system to test the feasibility of using the PLSRS to monitor pulse signals in blood vessels. However, for applications involving biological monitoring, high actuation temperatures pose safety hazards. To address this issue, we employed composition tuning to lower the *Tg* of the SMP and the nematic‐to‐isotropic transition temperature (*T_NI_*) of the LCE to approximately 39°C (Figure S15a). Infrared thermal image (Figure S15b) indicates that the maximum surface temperature of this low‐actuation‐temperature PLSRS during deformation is below the thermal damage threshold for biological tissues [48, 49]. Furthermore, to obtain high‐fidelity signals, the PLSRS must maintain stable contact with the blood vessel. The shape‐locking function of the PLSRS allows it to maintain a stable contacting state without power (Figure 4e). Since the actual monitoring is conducted after power‐off and cooling following the bending deformation, this fundamentally eliminates the risk of thermal injury while simultaneously avoiding motion artifacts introduced by continuous actuator tremor. This zero‐energy locking not only reduces energy consumption but also ensures signal stability. Experimental results show that the PLSRS can accurately capture the frequency and intensity characteristics of pulse waveforms (Figure 4f and Movie S7), with measurement accuracy comparable to commercial force sensors.

The robust actuation capability and stiffness‐tuning characteristics of the PLSRS make it an ideal modular architecture for constructing multifunctional soft robots. Addressing the limitation of traditional land‐air amphibious robots relying on bulky rigid motors for mode switching [50, 51], we developed a PLSRS‐based small‐scale land‐air robot (12.5 cm × 12.5 cm) (Figure 4g). The robot integrates two types of PLSRS: Type‐I PLSRS is used to adjust the thrust direction of the propellers, enabling configuration reconfiguration from rotor flight mode (Aerial State I) to wheeled rolling mode (Ground State II); Type‐II PLSRS is used for the morphological regulation of the landing gear (Figure S16 and Movie S8). This design validates the capability of the PLSRS to achieve configuration retention and multimodal locomotion switching in unstructured environments.

### 2.5 AI‐Enabled Bio‐Inspired Flight: From Wind Speed Sensing to Adaptive Control

**FIGURE 5:**
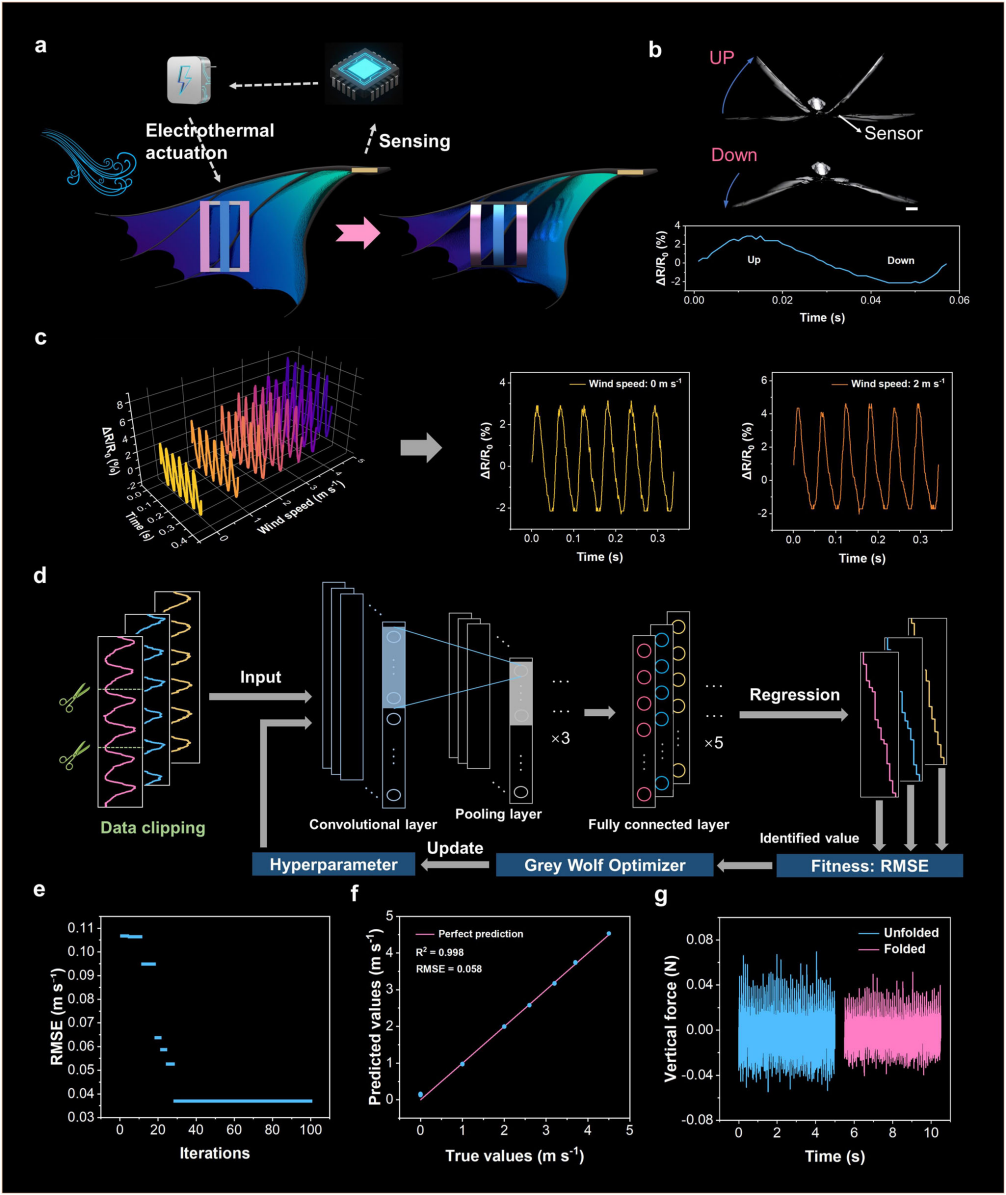
AI‐enabled flapping‐wing robot system for aeroelastic wind sensing and adaptive closed‐loop control. (a) Schematic illustration of the closed‐loop control system integrating sensing, decision‐making, and actuation. (b) Principle of wind speed sensing based on aeroelastic deformation of the wing spar. Scale bars: 2 cm. (c) Electrical signal response characteristics of sensors under different wind speeds. (d) 1D‐CNN architecture for feature extraction from time‐series signals. (e) Hyperparameter optimization process using GWO. (f) Wind speed prediction performance of the optimized model. (g) Lift generated by the flapping‐wing robot before and after wing folding.

Inspired by the biological mechanism where birds rely on mechanoreceptors to sense airflow and regulate wingspan to adapt to complex wind conditions [52], we integrated the PLSRS into a commercial flapping‐wing robot to construct a closed‐loop adaptive control system driven by deep learning‐based perception (Figure 5a). This system aims to address the critical challenge of balancing lift and structural safety for small‐scale aerial vehicles in unstructured airflow environments.

To endow the vehicle with a bio‐inspired wind sensing capability, we conformally attached the crack‐based sensor to the wing spar. During flapping flight, the interaction between the wing and airflow induces aeroelastic deformation. Since the crack‐based sensor is tightly bonded to the wing surface, this mechanical deformation is directly transferred to the sensor, causing the metal micro‐cracks to dynamically open and close according to the degree of deformation, thereby generating resistance fluctuation signals (Figure 5b). The total strain on the wing is a superposition of inertial and aerodynamic loads. As shown in Figure 5c, at a fixed flapping frequency, the resistance signal exhibits a fundamental periodicity synchronous with the mechanical flapping motion, which is primarily driven by inertial loads [53]. While inertial loads dominate the periodic amplitude, aerodynamic loads induce baseline shifts and local variations. When the wind speed increases (e.g., from 0 to 2 m/s), the altered aerodynamic loads exerted on the wing [54] lead to an upward shift of the signal baseline as well as an increase in peak‐to‐peak amplitude. This signal characteristic modulated by wind speed empowers the system to identify environmental wind speed solely through resistance changes.

However, while these macroscopic trends provide a physical basis for sensing, the actual aeroelastic signals during flapping flight are non‐linear and coupled with high‐frequency mechanical vibrations, motor noise, and transient turbulent fluctuations [55]. Extracting hidden aerodynamic features from such noisy time‐series data is challenging. Addressing this high complexity, traditional linear regression models are insufficient to achieve accurate decoupling. To this end, we developed a 1D convolutional neural network (1D‐CNN) architecture to automatically extract wind speed features from time‐series resistance signals (Figure 5d). Compared to fully connected networks, the 1D‐CNN possesses lower computational complexity, making it highly suitable for real‐time processing in embedded systems. To further enhance the prediction accuracy and generalization capability of the model, we introduced the grey wolf optimizer (GWO) to perform a global search for hyperparameters such as learning rate and kernel size (Table S2). The training process and evaluation results confirm the high fidelity of this sensing system. As shown in Figure 5e, after global optimization over 100 epochs, the root mean square error (RMSE) of the optimal model rapidly converged from an initial 0.107 to 0.037 m s^−^^1^, demonstrating the efficiency of the GWO algorithm in hyperparameter space search. To verify the necessity of introducing a deep learning model, we compared the 1D‐CNN with traditional linear regression (LR) and support vector regression (SVR) (Figure S17a). The results show that the LR model failed to capture the non‐linear features of the aeroelastic signals, with an RMSE as high as 2.059 m s^−^^1^. Even the SVR model, which possesses non‐linear fitting capabilities, yielded an RMSE (0.106 m s^−^^1^) nearly three times higher than that of the optimized 1D‐CNN (0.037 m s^−^^1^). Building on this, to validate the generalization capability of the model, we applied the trained network to an independent test set. The scatter plot of predicted versus actual wind speeds (Figure 5f) shows strong consistency, with a coefficient of determination (R^2^) as high as 0.998 and a final test set RMSE of only 0.058 m s^−^^1^. Further error statistical analysis indicates excellent robustness and precision, with an overall median error of only ‐0.007 m s^−^^1^, and the middle 50% of errors contained within the interval [‐0.025, 0.038] tightly centered around zero, indicating the absence of significant systematic bias. Box plot analysis of absolute errors grouped by wind speed (Figure S17b) further reveals that the model achieves optimal performance at a wind speed of 2.0 m s^−^^1^, with a median absolute error as low as 0.007 m s^−^^1^. Notably, among all 287 time‐series test samples, only 6 outliers were identified (accounting for only 2.09%). These data demonstrate the stability of the 1D‐CNN model in processing complex aeroelastic signals.

Based on precise wind speed sensing, we constructed a closed‐loop control system for autonomously regulating wing area (Movie S9). Its control strategy follows aerodynamic principles: Lift *L* is defined by the formula $L=\frac{1}{2}\;\rho \;v^{2}\;S\;C_{L}$, where *ρ* is the air density, *v* is the relative wind speed, *S* is the wing area, and *C_L_* is the lift coefficient [56]. At low wind speeds, the vehicle maintains full wingspan to maximize wing area *S*, thereby generating sufficient lift; at high wind speeds, to avoid excessive aerodynamic loads causing structural fatigue or excess lift inducing attitude instability, the system drives the PLSRS to contract and fold the wing (maximum folded area up to 25%, Figure S18). Notably, the zero‐energy locking characteristic of the PLSRS allows the vehicle to maintain the configuration without consuming additional electrical energy after reaching the target folded morphology, which is crucial for small‐scale vehicles limited by battery capacity. Experiments show that this variable‐configuration strategy effectively reduces lift at high wind speeds (Figure 5g). This system successfully demonstrates how the PLSRS‐enabled soft robot achieves quasi‐static or low‐frequency configuration adaptation through the fusion of physical intelligence (structural morphing, locking, and proprioception) and computational intelligence (intelligent perception and optimization).

## 3 Conclusions

In summary, we have established a robust, versatile, and integrable framework for constructing active and programmable shape‐morphing soft matter systems based on addressable actuation and strain‐constraint mechanisms. By strategically introducing localized strain mismatch via conductive constraint strips, this framework effectively circumvents the limitations of traditional microstructural manipulation techniques, offering a facile and efficient pathway for constructing complex 3D geometries. The integration of an analytical model with an inverse design strategy not only enables quantitative tuning of local curvature but also achieves high‐fidelity reconstruction of complex profile surfaces with spatially non‐uniform curvatures through a spatial discretization approach.

At the material and device level, we developed the PLSRS by synergistically integrating an LCE actuation layer, an SMP variable‐stiffness layer, and a crack‐based sensing layer. This system addresses the energy consumption bottleneck associated with shape retention in traditional soft actuators and endows the system with intrinsic proprioception capabilities. At the system application level, we integrated the PLSRS as an intelligent architecture within a flapping‐wing robot, establishing a closed‐loop adaptive control driven by deep learning‐based perception. Experimental results demonstrate that, through the deep fusion of physical intelligence (structural morphing, locking, and proprioception) and computational intelligence (intelligent perception and optimization), the system achieves efficient wind speed recognition and autonomous configuration adaptation in unstructured airflow environments.

Despite the aforementioned progress, there remains room for further optimization of the proposed framework. First, regarding dynamic response and thermal management, the reliance on Joule heating and natural cooling creates coupled challenges involving dynamic response, spatial footprint, and thermal safety. Inherently, natural cooling restricts the actuation frequency. Although we reduced the heating and cooling times to 1.9 s and 3.9 s by optimizing the working temperature window and the applied current (Figure S19), the system remains unsuitable for high‐frequency continuous dynamic control. Furthermore, to ensure the independent actuation of different materials and the thermal safety of the attached structures, we employed air gaps for spatial thermal decoupling. However, this inevitably increases the volume of the device and poses global heat diffusion challenges in enclosed spaces. To systematically resolve these issues in the future, a multi‐faceted strategy is required: (a) Replacing the air gaps with ultra‐thin thermal insulators (e.g., polyimide aerogel films [57]) and integrating directional heat spreaders (e.g., anisotropic graphene films [58]) to minimize the spatial footprint and enhance heat dissipation. (b) Incorporating high‐thermal‐conductivity fillers (e.g., liquid metal) or active microfluidic channels to accelerate cooling. (c) Employing composition tuning to customize the phase transition and actuation temperatures, thereby adapting to different application scenarios and alleviating thermal management pressures from the source. Second, regarding remote control, the current closed‐loop control relies on wired connections for power supply, sensor data acquisition, and computation, which limits the mobility of the soft robots. Achieving a fully untethered autonomous system requires future advancements across its core functional modules. In terms of actuation and power, lightweight and flexible batteries can be integrated onboard to eliminate power tethers. Alternatively, the conductive constraint strips can be substituted with photothermal or magneto‐thermal materials (as preliminarily demonstrated in Figure 1b) to enable remote actuation. In terms of sensing and computation, an intermediate step to untether the sensory feedback loop involves integrating onboard wireless communication modules to transmit data to an external device. However, to achieve true autonomy, the ultimate improvement requires transitioning from centralized processing to edge computing. By deploying the trained 1D‐CNN model directly onto an onboard microcontroller, the soft robot can process sensor data locally and execute adaptive control commands entirely onboard, ultimately eliminating the reliance on both physical tethers and external computing devices.

Overall, this work not only expands the application boundaries of stimuli‐responsive smart materials but also provides a robust and scalable solution for future autonomous soft robots, wearable electronics, and biomedical devices equipped with environmental perception and adaptive capabilities.

## 4 Experimental Section

### 4.1 Materials

1,4‐bis‐[4‐(6‐acryloyloxyhexyloxy)benzoyloxy]‐2‐methylbenzene (RM82) and 4‐(4‐((6‐(acryloyloxy)hexyl)oxy)phenyl 4‐((6‐(acryloyloxy)hexyl)oxy)benzoate (C6BAPE) were purchased from Shijiazhuang Yesheng Chemical Technology Co., Ltd. 3,6‐dioxa‐1,8‐octanedithiol (DODT), pentaerythritol tetrakis (3‐mercaptopropionate) (PETMP), and dipropylamine (DPA) were obtained from TCI. 2,2‐Dimethoxy‐2‐phenylacetophenone (I‐651) was purchased from BASF. Epoxy monomer E44 was purchased from China Petrochemical Corporation. 1,3‐Cyclohexanedimethanamine (1,3‐BAC) was purchased from Aladdin Bio‐Chem Technology Co., Ltd. The curing agent JEFFAMINE D230 was purchased from Huntsman Corporation. All chemicals were used as received.

### 4.2 Fabrication of Shape‐Morphing Structures

The mixture was formulated by combining RM82 and DODT in a molar ratio of 1.83:1, and DODT and PETMP in a molar ratio of 3:1, with the addition of 3 wt.% of I651, to produce a high‐*T_NI_* LCE. The mixture was formulated by combining RM82 and C6BAPE in a 1:9 molar ratio and DODT and PETMP in a 3:1 molar ratio to produce a low‐*T_NI_* LCE. The mixture was dissolved in dichloromethane. After thorough mixing, a catalytic amount of DPA was added to the solution. The mixture was rapidly transferred into a custom‐made Teflon mold (9.0 cm × 9.0 cm × 0.5 cm). The pre‐crosslinked LCE film was formed after 4 h at room temperature. The pre‐crosslinked film was stretched to 50% strain and subsequently irradiated under ultraviolet light for 120 s to obtain a fully oriented high‐*T_NI_* LCE film, whereas a low‐*T_NI_* LCE film requires 24 h of standing at room temperature. The LCE film was then cut into desired dimensions, and constraint strips were bonded onto its surface using ultraviolet light‐curing adhesive according to the designed size and layout.

### 4.3 Fabrication of SMPs

Epoxy monomer E44 and curing agent 1,3‐BAC were mixed in a beaker and placed in a vacuum oven for degassing. By replacing 1,3‐BAC with D230, an SMP with a lower *Tg* was prepared (with a molar ratio of E44 to D230 of 1:1). The mixture was then poured into a Teflon mold (9.0 cm × 9.0 cm × 0.5 cm). The SMP was obtained by curing the mixture according to the following protocol: 100°C for 2 h, followed by 130°C for 2 h.

### 4.4 Fabrication of Crack‐Based Sensor

A 7.5 µm‐thick polyimide (PI) film (3 M, USA) was cut to the desired dimensions. Subsequently, a 50 nm‐thick chrome (Cr) layer and a 20 nm‐thick gold (Au) layer were sequentially deposited onto its surface. The metallized PI film was stretched to 2% strain (at a tensile rate of 10 mm min^−^^1^) to generate micro‐cracks.

### 4.5 Characterization of Sensing Performance

Sensitivity and hysteresis: The sensor's response to bending was characterized by fixing one end of the sensor and controlling the bending angle of its free end. The corresponding steady‐state current was recorded at each incremental angle. The average sensitivity was determined by performing a linear fit on the current‐versus‐angle data across the primary operational range and taking the slope of this fitted line. Hysteresis was quantified by measuring the maximum difference in the current output during a complete loading‐unloading cycle. Dynamic response: The dynamic response was evaluated by subjecting the sensor to a rapid, step‐like bending. The rise time (10%–90% of the total signal change) was determined from the transient current response curve, which was recorded at a sampling rate of 1 kHz.

### 4.6 Simulation and Computation

The optimization for the inverse design strategy was numerically solved using Wolfram Mathematica (utilizing its built‐in non‐linear local optimization algorithms, such as the FindMinimum function). The FEA for predicting the morphing behaviors and verifying the thermal decoupling mechanism was conducted using the commercial software COMSOL Multiphysics.

### 4.7 General Characterizations

A power source (ITECH 6332A) was used to offer power. A high‐precision source meter (KEITHLEY 2450) was used to monitor electrical signals. Photos and videos of the actuation experiments were recorded by a digital camera (Canon, EOS 80D(W)). Differential scanning calorimetry (DSC) measurement was carried out by a TA Instruments DSC250 with a heating rate of 10°C min^−1^ in a nitrogen atmosphere.

## Author Contributions

K.L. and J.‐L. conceived the initial idea and designed the experiments. K.L. performed the experiments, the finite‐element modelling and theoretical study and analyzed the experimental data. K.L., P.X., R.S., B.L., and R.G. contributed to data analysis. J.‐L. contributed to funding acquisition, provision of resources, and writing – review and editing.

## Conflicts of Interest

The authors declare no conflicts of interest.

## Supporting information




**Supporting File 1**: advs76241‐sup‐0001‐SuppMat.pdf.

**Supporting File 2**: advs76241‐sup‐0002‐MovieS1.mp4.

**Supporting File 3**: advs76241‐sup‐0003‐MovieS2.mp4.

**Supporting File 4**: advs76241‐sup‐0004‐MovieS3.mp4.

**Supporting File 5**: advs76241‐sup‐0005‐MovieS4.mp4.

**Supporting File 6**: advs76241‐sup‐0006‐MovieS5.mp4.

**Supporting File 7**: advs76241‐sup‐0007‐MovieS6.mp4.

**Supporting File 8**: advs76241‐sup‐0008‐MovieS7.mp4.

**Supporting File 9**: advs76241‐sup‐0009‐MovieS8.mp4.

**Supporting File 10**: advs76241‐sup‐0010‐MovieS9.mp4.

## Data Availability

The data that support the findings of this study are available in the supplementary material of this article.
