# Supplementary material for: A Versatile‐Designable Framework for Active and Programmable Shape‐Morphing Soft Matter Systems: From Inverse Design to Closed‐Loop Control
Source: Adv Sci (Weinh). 2026 Jun 22:e76241. Online ahead of print. doi: 10.1002/advs.76241 (PMC13336906; doi:10.1002/advs.76241)
Supplement: Supplementary file 1 — Supporting File 1: advs76241‐sup‐0001‐SuppMat.pdf. [file ADVS-9999-e76241-s005.pdf]

Supporting Information

A Versatile-designable Framework for Active and Programmable Shape-Morphing Soft Matter Systems: From Inverse Design to Closed-Loop Control

Kai Liu[1,2,3], Peiling Xie[1,2,3], Ruitong Song[1,2,3], Banghan Liu[1,2,3], Rui Guo[1,2,3], Jiu-an Lv[2,3]*

[1] School of Materials Science and Engineering, Zhejiang University, Hangzhou 310027, China.

[2] Key Laboratory of 3D Micro/Nano Fabrication and Characterization of Zhejiang Province, School of Engineering, Westlake University, 18 Shilongshan Road, Hangzhou 310024, Zhejiang Province, China.

[3] Institute of Advanced Technology, Westlake Institute for Advanced Study, 18 Shilongshan Road, Hangzhou 310024, Zhejiang Province, China.

Correspondence and requests for materials should be addressed to J. L. (lvjiuan@westlake.edu.cn)

Contents:

1. Supplementary Text S1.

2. Supplementary Figures S1 to S19.

3. Supplementary Tables S1 and S2.

Supplementary Text

Text S1 Theoretical model of shape morphing induced by strain constraint

In a composite structure consisting of two material layers, comprising a thermally contractile liquid crystal elastomer (LCE) substrate and constraint strips fabricated from a high-modulus material, a strain mismatch arises upon temperature change. Specifically, the LCE undergoes contraction while the constraint strips remain dimensionally stable. This interlayer strain mismatch generates an internal bending moment, driving the integral bending of the LCE into a tubular configuration. Figure S1 shows the coordinate system and key geometric parameters. The origin of the coordinate system (z = 0) is defined at the geometric mid-plane of the LCE layer. ε_{yy} denotes the contraction strain of the LCE along the y-axis, and ε_{yym} represents the contraction strain of the bilayer structure's neutral plane along the y-axis. Analogous to beam bending theory, we assume a plane stress condition where the normal stress along the thickness direction is negligible, i.e.,

$$\sigma_{zz} = 0$$

For a tubular configuration characterized by curvature κ, the contraction strain of the LCE along the y-axis is given by:

$$\varepsilon_{yy} = \kappa\, z + \varepsilon_{yym} - \alpha_s\,(T - T_0)$$

The elastic energy of a representative unit of the LCE substrate is given by:

$$U_s = \int_{-\frac{1}{2}h_s}^{\frac{1}{2}h_s} \frac{1}{2}\, l_s\, W_s\, \sigma_{ij}\, \varepsilon_{ij}\, dz = \frac{1}{24}\frac{E_s\, W_s\, l_s\, h_s}{1 - \upsilon_s^2}\left[12\,\left(T_0\, \mathrm{\alpha_s} - T\, \mathrm{\alpha_s} + \varepsilon_{yym}\right)^2 + h_s^2\, \kappa^2\right]$$

For the constraint strip:

$$\varepsilon_{yy} = \kappa\, z + \varepsilon_{yym} - \alpha_r\,(T - T_0)$$

The elastic energy of a representative unit of the constraint strip is given by:

$$U_r = \int_{\frac{1}{2}h_s}^{\frac{1}{2}h_s+h_r} \frac{1}{2}\, l_r\, W_r\, \sigma_{ij}\, \varepsilon_{ij}\, dz$$

$$= -\frac{1}{6}\frac{E_r\, W_r\, l_r}{\kappa\,(1-\upsilon_r^2)}\left[\left(T_0\, \alpha_r - T\, \alpha_r + \varepsilon_{yym} + \frac{h_s\, \kappa}{2}\right)^3 - \left(T_0\, \alpha_r - T\, \alpha_r + \varepsilon_{yym} + h_r\, \kappa + \frac{h_s\, \kappa}{2}\right)^3\right]$$

where υ represents the Poisson's ratio. According to the principle of minimum potential energy, the equilibrium state of the system corresponds to the minimization of the total elastic energy:

$$\frac{\partial(U_s + U_r)}{\partial\kappa} = 0$$

$$\frac{\partial(U_s + U_r)}{\partial\varepsilon_{yym}} = 0$$

By simultaneously solving the aforementioned system of equations, the expressions for the curvature κ and the contraction strain ε_{yym} can be derived:

$$\kappa = \frac{6\, E\, W\, l}{h_s}\frac{h\,(1+h)\,(T-T_0)\,(\alpha_r-\alpha_s)}{1+2\, E\, W\, l\, h\,(2+3\,h+2\,h^2)+\, E^2\, W^2\, l^2\, h^4}$$

$$\varepsilon_{yym} = \frac{(T-T_0)\{E^2\, W^2\, l^2\, h^4\, \alpha_r + \alpha_s + E\, W\, l\, h\,[\alpha_r + \alpha_s(3+6\,h+4\,h^2)]\}}{1+2\, E\, W\, l\, h\,(2+3\,h+2\,h^2)+\, E^2\, W^2\, l^2\, h^4}$$

Supplementary Figures

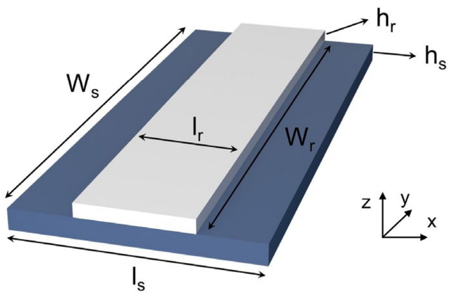

Figure S1. Schematic illustration of geometric parameters used in the analytical model.

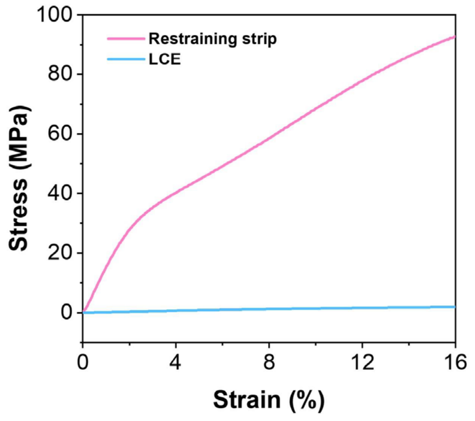

Figure S2. Elastic modulus contrast between the LCE substrate and the constraint strip.

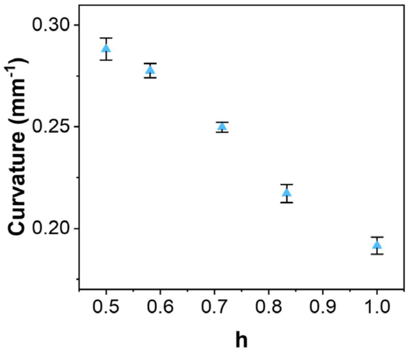

Figure S3. Effect of relative thickness on curvature. Data are presented as mean ± SD (*N* = 3).

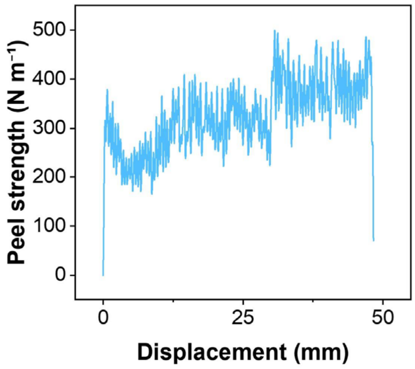

Figure S4. Interfacial peel strength test between the LCE substrate and the constraint strip.

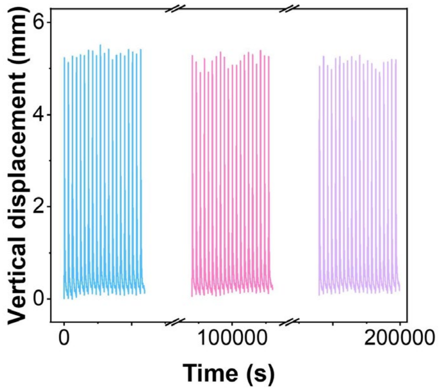

Figure S5. Cyclic actuation test of the morphing system.

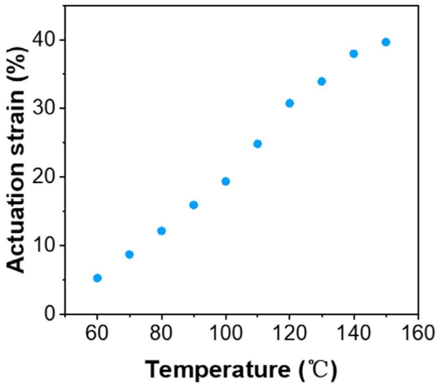

Figure S6. The actuation strain of LCE.

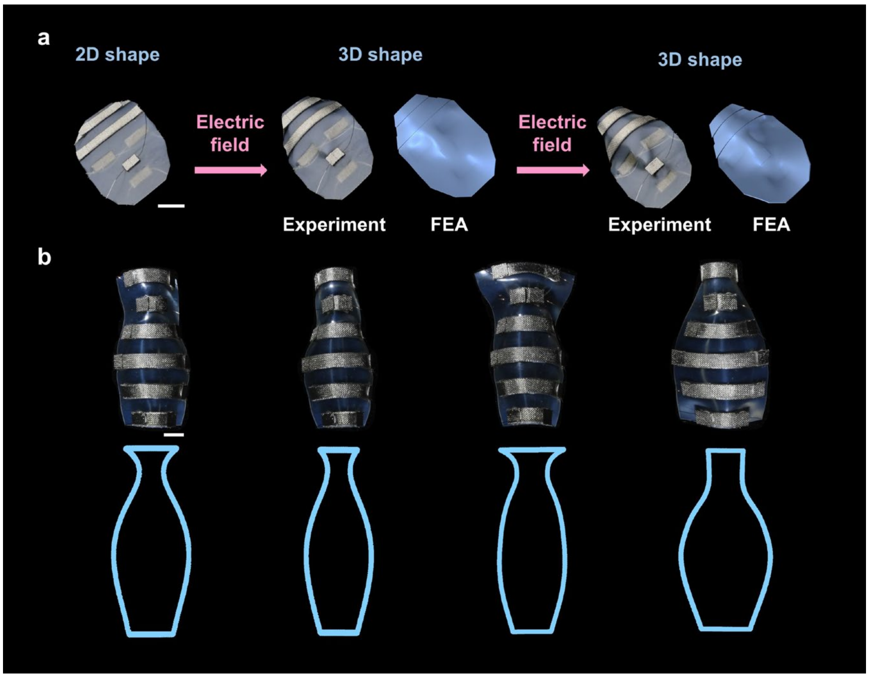

Figure S7. Construction of complex surfaces and multimodal shape reconfiguration. a) Demonstration of complex human face profile morphing. Scale bars: 7 mm. b) Multimodal shape reconfiguration enabled by independent addressing capability. Scale bar: 4 mm.

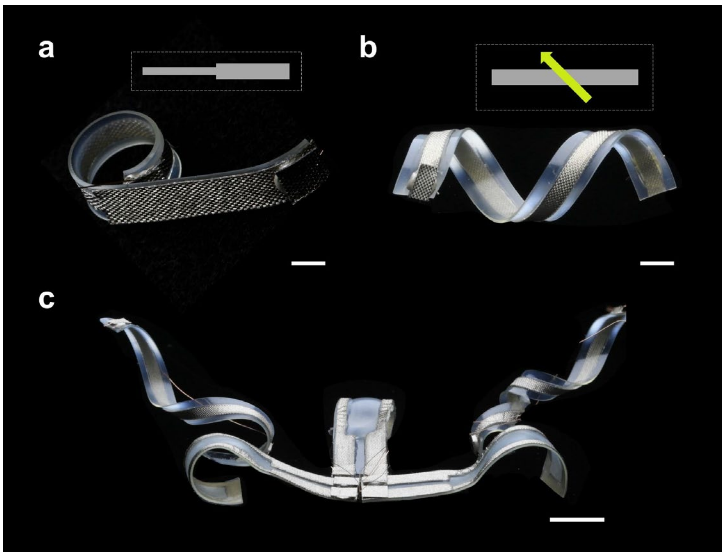

Figure S8. Complex morphing based on strain constraint. a) Effect of constraint strip width on bending curvature induced by coupled mechanical and electrothermal effects. The inset indicates the width difference of the constraint strips (2 mm and 4 mm). Scale bar: 4 mm. b) Torsional actuation enabled by combining strip design with LCE orientation. The inset illustrates the angular relationship between the constraint strip and the LCE orientation, where the green arrow indicates the LCE orientation, the grey rectangle represents the constraint strip, and the angle between the LCE orientation and the long axis of the constraint strip is 45°. Scale bar: 4 mm. c) Morphing of a bio-inspired flower structure. Scale bar: 10 mm.

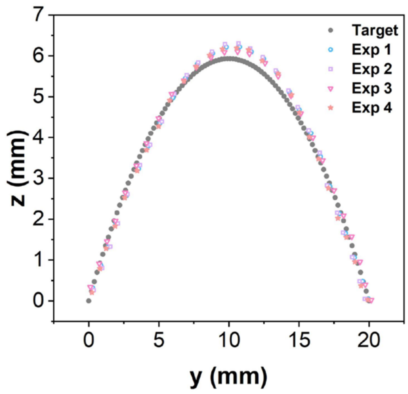

Figure S9. A comparison of the deformation profiles of four independent samples used to reconstruct a spatially non-uniform curved surface.

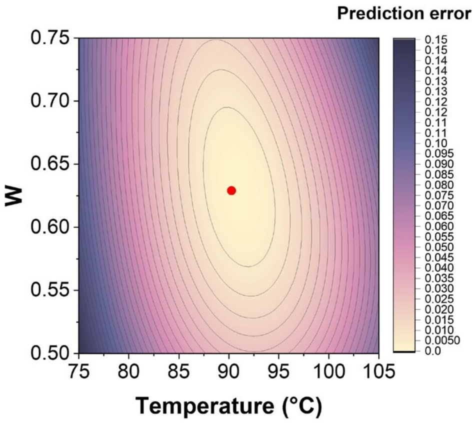

Figure S10. Perturbation analysis and contour plot of the objective function.

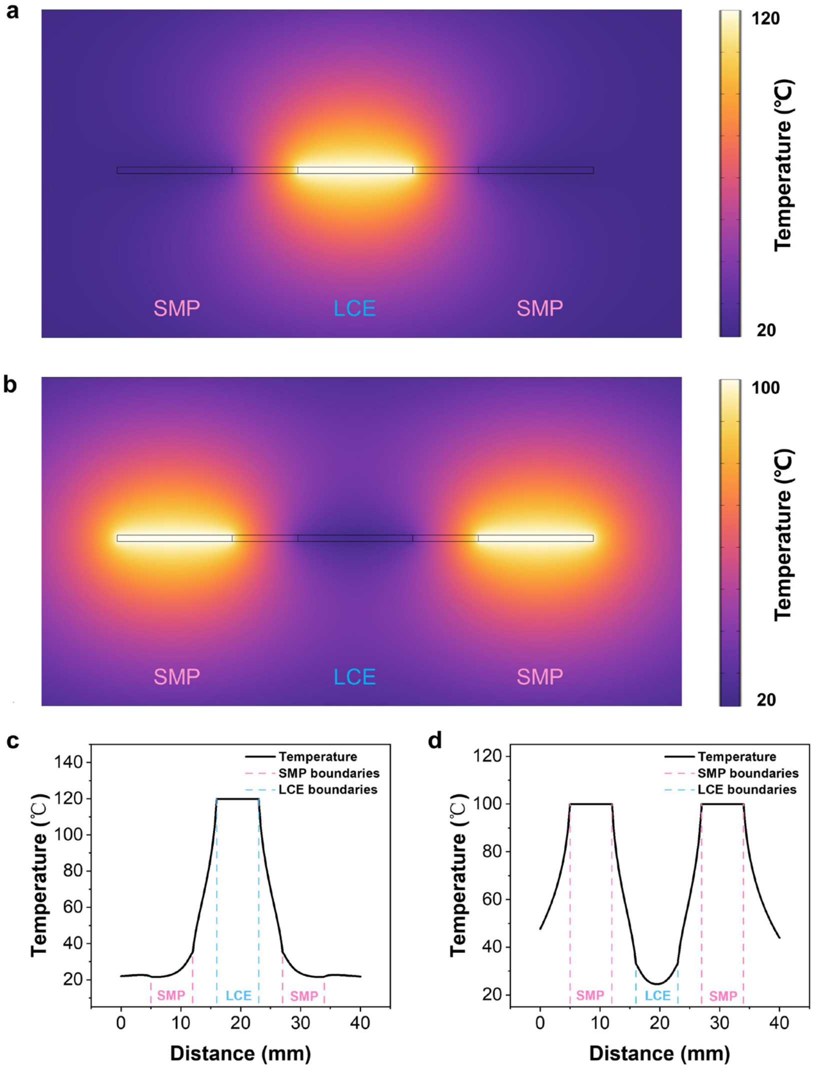

Figure S11. Finite Element Analysis (FEA) verification of the thermal decoupling mechanism. a) Simulated temperature distribution of the PLSRS cross-section along the transverse direction when heating a single LCE strip. b) Simulated temperature distribution of the PLSRS cross-section along the transverse direction when heating two SMP strips. c) Cross-sectional temperature profile of the PLSRS along the transverse direction when heating a single LCE strip. d) Cross-sectional temperature profile of the PLSRS along the transverse direction when heating two SMP strips.

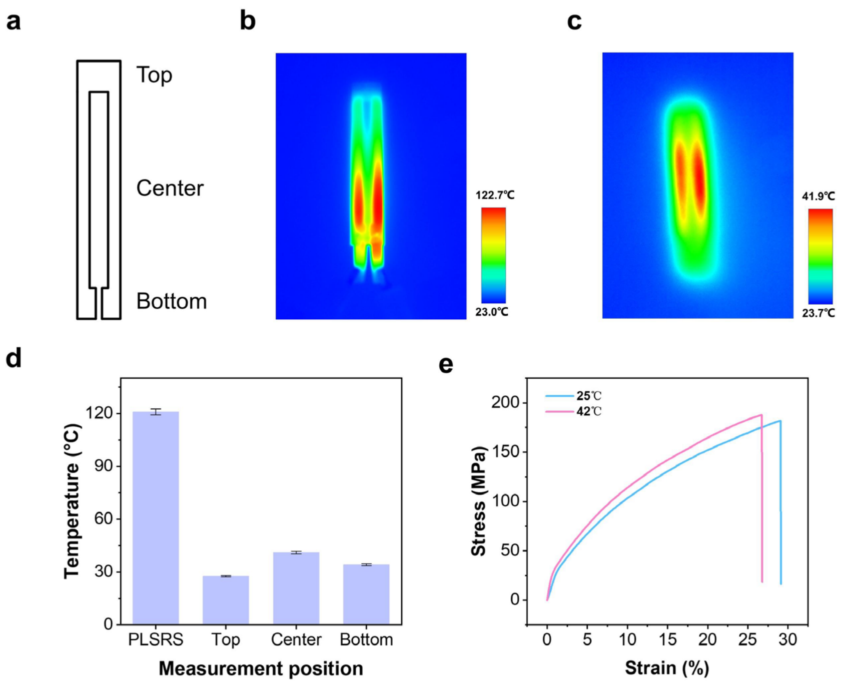

Figure S12. Experimental verification of the thermal decoupling mechanism. a) Schematic illustration of the conductive constraint layer design. b) Infrared thermal image of the conductive constraint layer during electrical heating. c) Infrared thermal image of the attached material during electrical heating. d) Temperatures at different measurement positions on the attached material and the PLSRS. e) Uniaxial stress-strain curves of the wing film at different temperatures.

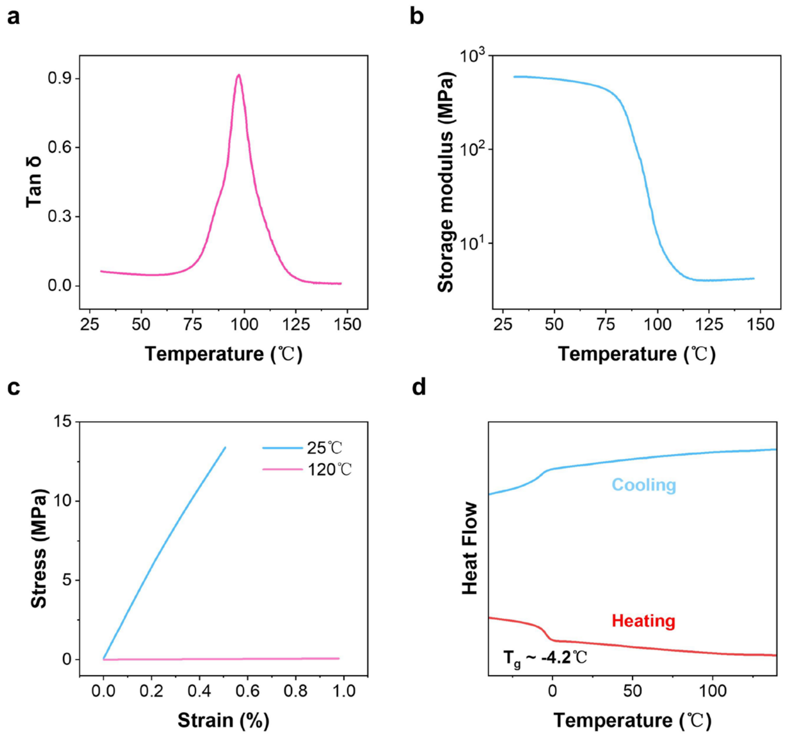

Figure S13. Performance characterization of the shape memory polymer (SMP) and LCE. a) Temperature dependence of the loss tangent (tan *δ*) for the SMP. b) Temperature dependence of the storage modulus for the SMP. c) Uniaxial stress-strain curves of the SMP at 25 °C and 120 °C, corresponding to moduli of 2.68 GPa and 7.28 MPa, respectively. d) Differential scanning calorimetry (DSC) curves of the LCE.

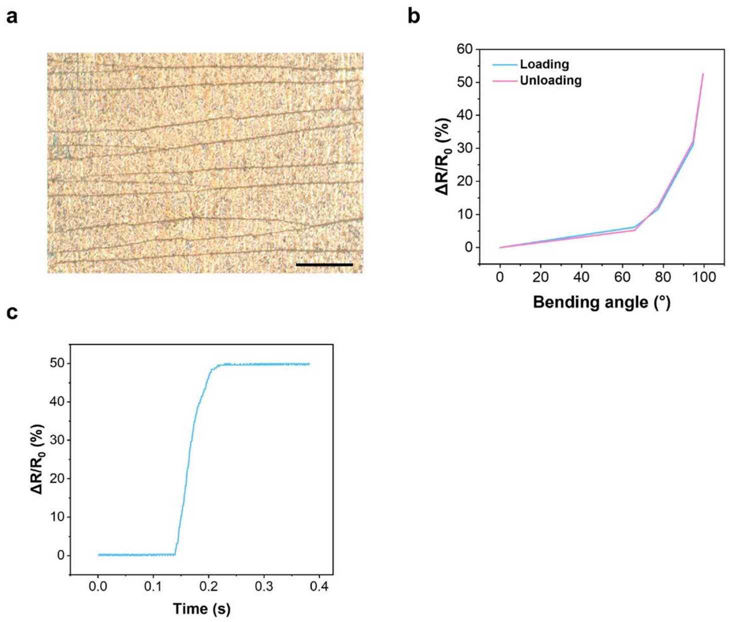

Figure S14. Sensing performance of the crack-based sensor. a) Image of metal cracks on the sensor surface. Scale bar: 100 μm. b) Hysteresis performance of the sensor. c) Dynamic response performance of the sensor.

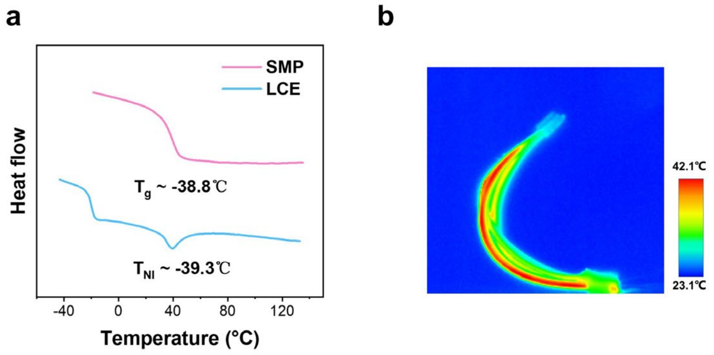

Figure S15. Verification of the low-actuation-temperature PLSRS. a) DSC curves of the low-T_{NI} LCE and the low-Tg SMP. b) Infrared thermal image of the low-actuation-temperature PLSRS during bending.

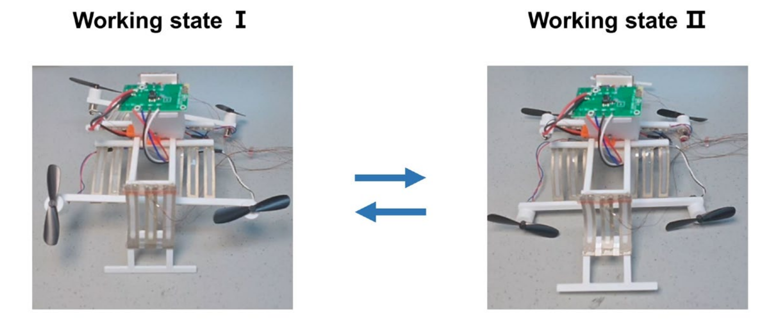

Figure S16. The morphological regulation of the landing gear between State I and State II.

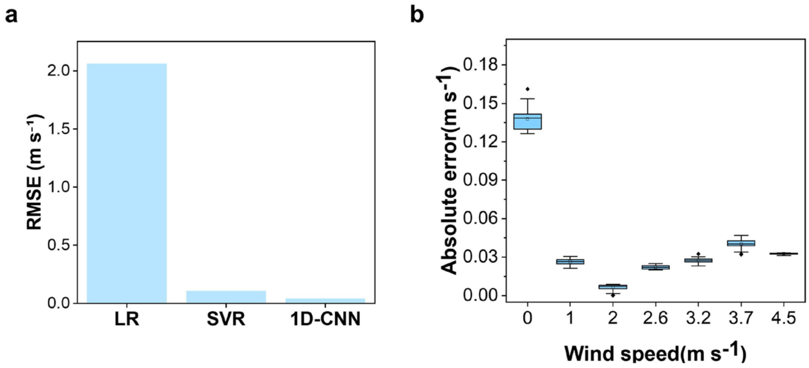

Figure S17. Statistical error analysis of the models. a) Error comparison among different models. b) Box plot of the absolute errors of the 1D-CNN model.

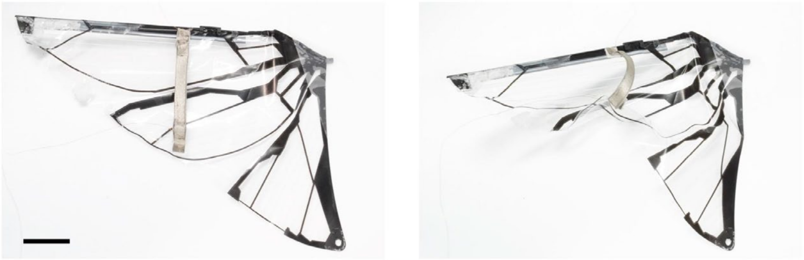

Figure S18. Demonstration of the maximum folding area of the wing. Scale bars: 2 cm.

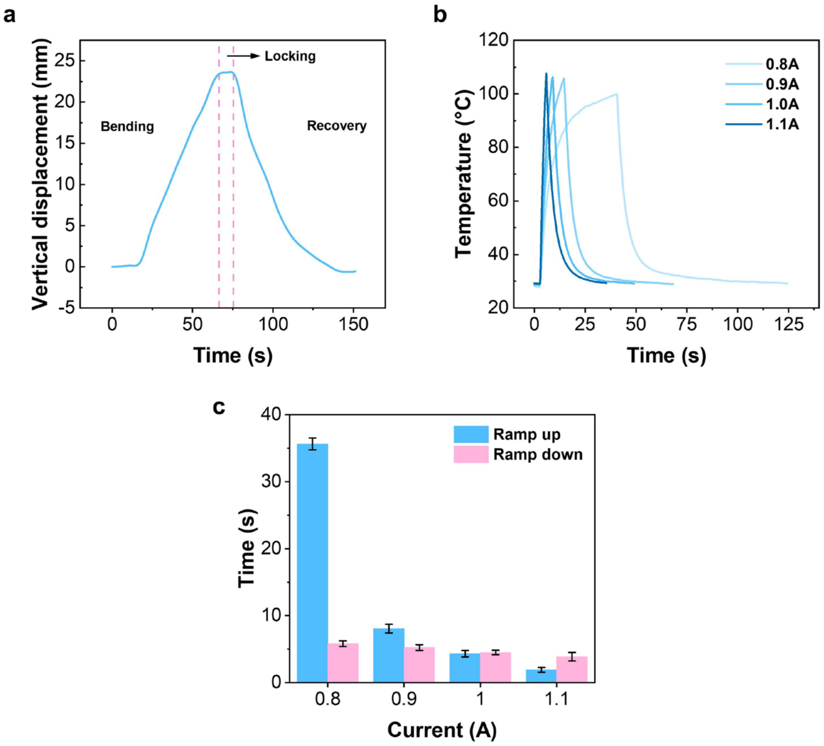

Figure S19. Quantitative characterization of the two-way shape morphing and tunable thermal response time of the device. a) The vertical actuation displacement-time response curve during a complete two-way morphing cycle. b) Temperature-time curves of the device during heating and natural cooling at different currents. c) Ramp-up time (heating from 50 °C to 100 °C) and ramp-down time (cooling from 100°C to 50°C) of the device at different currents.

Supplementary Tables

Table S1. Optimized parameters for vase profile reconstruction.

Constraint strip	W	T (°C)
1	0.63	90
2	0.32	135
3	0.75	83
4	0.97	75
5	0.82	80
6	0.51	101

Table S2. The hyperparameters of the neural network for wind recognition.

Input layer	Parameter	Kernel size
Conv block 1	Kernel size	86
	Filters	360
	Pool size	91
	Pool stride	10
Conv block 2	Kernel size	43
	Filters	79
	Pool size	7
	Pool stride	2
Conv block 3	Kernel size	2
	Filters	63
	Pool size	1
	Pool stride	1
FC network	FC1 units	122
	FC2 units	61
	FC3 units	210
	FC4 units	29
	FC5 units	14
Training	Learning rate	0.0062
